# Associations between patient clinical characteristics and the presence of cytomegalovirus DNA in the bronchoalveolar lavage fluid of children with recurrent wheezing

**DOI:** 10.1186/s12879-018-3345-9

**Published:** 2018-09-10

**Authors:** Huiming Sun, Shuxiang Li, Yongdong Yan, Zhengrong Chen, Yuqing Wang, Chuangli Hao, Wei Ji

**Affiliations:** 1 Department of Respiratory Medicine, Children’s Hospital of Soochow University, No. 303, JingDe Road, Suzhou, Jiangsu China; 2 Department of Clinical Laboratory, Suzhou Hospital Affiliated to Nanjing Medical University, No. 26, Daoqian Road, Suzhou, Jiangsu China

**Keywords:** Bronchoalveolar lavage fluid, Cytomegalovirus, Asthma, Wheezing

## Abstract

**Background:**

This study aimed to investigate the occurrence of Cytomegalovirus (CMV) DNA in the Bronchoalveolar lavage fluid (BALF) of children with recurrent wheezing and to identify associations with certain patient clinical characteristics.

**Methods:**

In this cross-sectional study, pediatric patients (age < 36 months) admitted to Soochow University Hospital with recurrent wheezing (≥ 4 episodes of wheezing per year) were enrolled in the study. Cytomegalovirus DNA from their BALF was detected by real-time PCR. Subpopulations of blood immunoglobulins and T lymphocytes were quantified. The clinical characteristics of patients with and without BALF CMV DNA were compared. Comparisons of non-normally distributed continuous variables between groups were made using the Mann-Whitney U-test. Comparisons of frequency distributions were made using the Chi-squared test. Spearman’s rank correlation coefficient was used to evaluate correlations between the number of CMV DNA copies and continuous variables.

**Results:**

A total of 111 patients aged 4 to 36 months (median 14.0 (IQR 8.0–22.0) months) were enrolled on to the study. Cytomegalovirus DNA was detected in 51.4% of patients (*n* = 111) with recurrent wheeze and was more prevalent among those aged 12 to 36 months with a positive modified asthma predictive index (mAPI) (*n* = 38, median 23.5 (IQR 19.7–31.2) months) than in those of the same age group with a negative mAPI (*n* = 25, median 15.0 (IQR 13.0–19.0) months) (57.9% vs. 20.0%, *p* = 0.003). Bronchoalveolar lavage fluid CMV DNA copy number [median 7560 (IQR 1200–71,150) copies/mL] was positively correlated with the duration of hospitalization (*r* = 0.33, *p* = 0.013), and negatively correlated with patient age (*r* = − 0.41, *p* = 0.002) and the percentage of BALF eosinophils (*r* = − 0.38, *p* = 0.004).

**Conclusions:**

CMV infection or reactivation in children with recurrent wheeze is associated with certain clinical characteristics, including younger age and lower levels of BALF eosinophils. Higher CMV DNA copy numbers were associated with a longer duration of hospitalization. Further studies are needed to address whether specific antiviral treatment could be beneficial for BALF CMV positive patients.

## Background

Human cytomegalovirus (CMV) is a member of the herpesvirus family that is prevalent among adult populations of Westernized countries, where the infection rate ranges from 60 to 70% [1]. Most immunocompetent individuals with CMV infections experience mild symptoms or are asymptomatic. However, in those with immature immune systems, such as fetuses and young infants, or in those who are immunocompromised, acute CMV infection may produce a variety of symptoms including fever, malaise, enlarged lymph nodes, sore throat, muscle aches, loss of appetite, enlarged liver or spleen, and fatigue [2].

Recurrent episodes of wheezing are a major cause of morbidity in young children and are characteristically heterogeneous in their presentation and underlying causes [3]. Disease progression is variable in these patients, some will have only transient wheezing associated with recurrent respiratory viral infections, whereas others will have persistent asthma. The ability to predict subsequent school-age asthma from recurrent wheeze at preschool age has proven challenging. Since the performance of spirometry testing is not reliable before the age of five, the modified asthma predictive index (mAPI) is routinely used as a validated clinical model for predicting childhood asthma [4].

Several previous studies have reported an association between CMV infection and wheezing in immunocompetent infants [5–7]. However, none of these studies have specifically focused on CMV infection in patients with recurrent wheeze. Since CMV is a persistent virus that is shed for a long period of time after the primary infection, we hypothesized that immune reaction to latent CMV infection may promote bronchial inflammation in patients with recurrent wheeze.

The pathophysiology of recurrent wheeze in young children is unclear. Persistent bronchial inflammation was thought to play an essential role, but more recently it has been reported that the presence of CMV DNA in the blood of adult and elderly patients was associated with an increased risk of asthma, and that CMV DNA copy numbers correlated with certain asthma traits [8]. This suggests that CMV infection may play a role in the pathophysiology of recurrent wheeze. However, the presence of CMV DNA in a blood sample does not necessarily equate with clinical effects of CMV infection in the lung. Therefore, the present study was carried out to examine the association between presence of CMV DNA in the bronchoalveolar lavage fluid (BALF) and clinical characteristics of patients with recurrent wheeze.

## Methods

The study had a cross-sectional design and took place in Soochow University Hospital in the city of Suzhou, Jiangsu province, China, from May 2014 to December 2017. Data were collected prospectively during that period from patients admitted consecutively to our department with recurrent wheezing. From the prospectively collected data, children aged < 36 months who had been admitted to the hospital consecutively with recurrent wheezing were considered eligible for the study if they had experienced wheezing for ≥ 4 weeks despite treatment with bronchodilators, inhaled corticosteroids or at least 15 days of treatment with systemic corticosteroids, and had experienced more than four episodes of wheezing in the previous 12 months. Patients were excluded from the study if any of the following were applicable: born prematurely or birthweight < 2500 g; family history of smoking; airway structural abnormalities (e.g. malacia, bronchial stenosis); endobronchial tuberculosis; gastroesophageal reflux; congenital or acquired immunodeficiencies; foreign-body aspiration; congenital heart disease, or; neuromuscular disorder.

Patients meeting the above eligibility criteria were enrolled on the study after informed and signed consent was received from their legal guardians. Enrolled patients underwent FFB and were tested for BALF CMV DNA. Flexible fiberoptic bronchoscopy was performed in accordance with the hospital guidelines. The study protocols were approved by the Ethics Committee of the Children’s Hospital of Soochow University.

### Clinical definitions

Recurrent wheeze was defined as ≥4 episodes of wheeze within 1 year as documented in the patients’ medical records. A wheezing episode was defined as a respiratory episode with wheezing lasting for more than 1 day. The interval between two wheezing episodes was defined as a period of ≥7 days without respiratory symptoms.

A mAPI was considered positive when a patient had ≥4 wheezing episodes in a year while also exhibiting one of three defined major criteria (parental asthma, allergic sensitization to one or more aeroallergens, or physician-diagnosed dermatitis) or two of three defined minor criteria (wheezing unrelated to colds, peripheral blood eosinophils ≥4%, or allergic sensitization to milk, egg or peanuts) [4].

### Baseline data collection

The following clinical characteristics were obtained from all recruited patients through interviews and reviews of their medical records: age; gender; exclusive breastfeeding during the first 4 months of life; other children in the household; previous wheezing episodes and duration of hospitalization. Laboratory data were collected from peripheral blood samples which were taken on admission to the hospital: peripheral blood eosinophil count was determined using an automated hematology analyzer (XE-2100, Sysmex, Japan); counts of subpopulations of lymphocytes were determined by a Coulter Epics XL MCL Flow Cytometer (Beckman Coulter, USA) after labelling with the following fluorochrome-antibody combinations: anti-CD45-fluorescein isothiocyanate/anti-CD4-phycoerythrin/anti-CD8-phycoerythrin-Texas red/anti-CD3-phycoerythrin-cyanin 5.1; anti-CD45- fluorescein isothiocyanate/anti-CD16CD56-phycoerythrin/anti-CD19-phycoerythrin-Texas red/anti-CD3- phycoerythrin-cyanin 5.1; anti-CD45-fluorescein isothiocyanate; anti-CD19-phycoerythrin-Texas red; anti-CD23-allophycocyanin; and anti-CD3-phycoerythrin. All fluorochrome-antibodies were purchased from Immunotech (France). Cells stained with antibodies were analyzed by flow cytometry using EXPO32™ ADC software. A CD45/SS gating strategy was used for the identification of T-, B- and NK-cell populations in peripheral blood; total IgG, IgA and IgM levels were determined using an Olympus AU400 analyzer (Olympus, Japan), and; total IgE was determined in a subset of 36 patients using the ImmunoCAP system (Pharmacia Diagnostics, Sweden) in accordance with the manufacturer’s instructions. Skin prick testing was performed in a total of 75 patients to 18 common allergens (food allergens and aeroallergens), using allergen extracts and testing devices manufactured by Becton Dickinson (USA).

### Flexible fiberoptic bronchoscopy and bronchoalveolar lavage fluid testing

A flexible fiberoptic bronchoscope [Olympus CV260 (2.8 mm, 4.0 mm), Japan, or Fujinon EB-270P (3.6 mm), Japan] was chosen according to the age of the patient, and used to conduct FFB in accordance with previously described procedures [9]. During the procedure, BALF was collected for cell count analysis and CMV DNA detection.

### Cell counts

Differential cell counts were obtained using a modified version of Wright-Giemsa staining (Wright-Giemsa Stain, Baso Diagnostics Inc., China). At least 500 cells were examined by the same observer for each specimen. Data were reported as percentages of the total cell counts.

### Detection of CMV

The detection and quantification of the CMV DNA level was carried out by real-time PCR, using a diagnostic kit for the quantification of human CMV DNA (Sansure Biotech, China) and a LightCycler 480 system (Roche Applied Science, USA), according to the manufacturer’s instructions. The detection limit for the test was > 400 copies/mL.

### Statistical analysis

Statistical analyses were performed using SPSS software, version 21.0 (IBM SPSS). The Kolmogorov-Smirnov test was used for testing data for normality. Continuous variables with non-normal distributions were expressed as medians and interquartile ranges (IQR) (25th to 75th percentile). Comparisons between groups were made using the Mann-Whitney U-test. Spearman’s rank correlation coefficient was used to evaluate correlations between the number of copies of CMV DNA and the various continuous variables. Comparisons of frequency distributions were performed using the Chi-squared test. A test result was deemed statistically significant if *p* < 0.05. A sample size estimation was calculated based on a likely sample proportion having the tested trait (P) of 50% [5], with 95% confidence (α = 0.05) and a 10% margin of error of the estimate. The minimum required sample size according to these parameters and the formula n = $\frac{{\mathrm{Z}}_{\alpha /2}^2\mathrm{P}\left(1-\mathrm{P}\right)}{d^2}$, was *n* = 96.

## Results

A total of 111 patients aged 4 to 36 months (median 14.0 (IQR 8.0–22.0) months) were enrolled in the study. Of these, 50 patients had a positive mAPI and 61 had a negative mAPI.

**Fig. 1:**
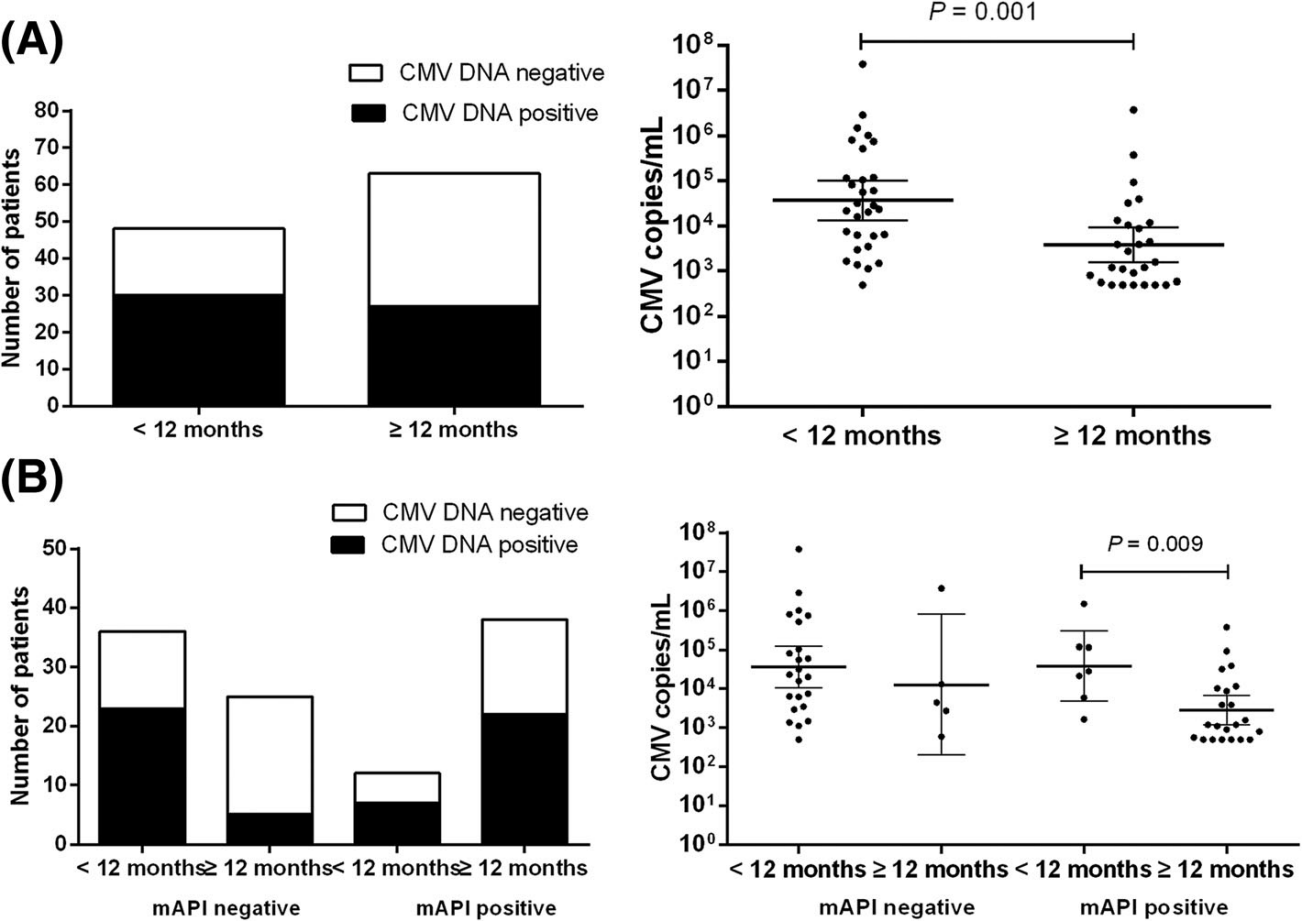
Analyzed CMV DNA in the BALF of 111 patients with recurrent wheezing; Soochow, 2014–2017. **a** Number of patients and their CMV load [median (IQR)] in patients aged < 12 months or patients aged 12 to 36 months; (**b**) Number of patients and their CMV load [median (IQR)] in patients aged < 12 months or 12 to 36 months with positive mAPI, and in patients aged < 12 months or 12 to 36 months with negative mAPI

Bronchoalveolar lavage fluid CMV DNA was detected in 51.4% of patients (*n* = 111). Test positivity was higher in patients aged < 12 months (*n* = 48, median 7.5 (IQR 5.2–9.0) months) than in those aged 12 to 36 months (*n* = 63, median 21.0 (IQR 16.0–29.0) months) (62.5% vs. 42.7%, *p* = 0.040) (Fig. 1a), and higher in patients aged 12 to 36 months with a positive mAPI (*n* = 38, median 23.5 (IQR 19.7–31.2) months) than in those aged 12 to 36 months with a negative mAPI (*n* = 25, median 15.0 (IQR 13.0–19.0) months) (57.9% vs. 20.0%, *p* = 0.003) (Fig. 1b). On the other hand, the median age of patients aged 12 to 36 months with a positive mAPI (*n* = 38) was higher than that of patients aged 12 to 36 months with a negative mAPI (*n* = 25) (23.5 (IQR 19.7–31.2) months vs. 15.0 (IQR 13.0–19.0) months, *p* < 0.001). The median BALF CMV DNA copy number was higher in patients aged < 12 months (*n* = 48) than in patients aged 12 to 36 months (*n* = 63) (median 26,050 (IQR 5340–219,750 copies/mL) vs. 1580 (560–11,700) copies/mL, *p* = 0.001) (Fig. 1a). After stratifying the enrolled patients according to their ages and mAPIs, we found that patients aged < 12 months with positive mAPI had higher BALF CMV DNA copy numbers than those aged 12 to 36 months with a positive mAPI (median 28,800 (IQR 5960–120,000) copies/mL vs. 1200 (500–10,725) copies/mL, *P* = 0.009) (Fig. 1b).

The clinical characteristics of patients identified as being BALF CMV positive and BALF CMV negative are presented in Table 1. Patients who were BALF CMV positive possessed a higher percentage of CD3^+^CD8^+^ T cells (median 24.3% (IQR 17.6–28.6) vs. 18.5% (15.6–26.2), *p* = 0.023) as well as a lower percentage of BALF eosinophils (median 0.0 (IQR 0.0–2.0) % vs. 0.5 (0.0–6.0) %, *p* = 0.027) than patients who were BALF CMV negative. The median duration of hospitalization was longer in patients who were BALF CMV positive (median 11.0 (IQR 9.0–13.2) days vs. 11.0 (8.0–13.0) days, *p* = 0.001).

**Table 1:** Clinical characteristics of 111 patients identified as BALF CMV positive or negative, Soochow, 2014–2017

Variable	BALF CMV positive (*n* = 57)	BALF CMV negative (*n* = 54)	*P-value*
Age, median (IQR), months	11.0 (6.0–21.5)	14.5 (9.7–23.0)	0.133
Male, n (%)	42 (73.7)	43 (79.6)	0.460
Breastfeeding, n (%)	35 (61.4)	30 (55.6)	0.532
≥ 2 children in household, n (%)	34 (59.6)	37 (68.5)	0.331
Length of hospitalization, median (IQR), days	11.0 (9.0–13.2)	11.0 (8.0–13.0)	0.001
Peripheral blood eosinophils, median (IQR), %	0.7 (0.3–1.3)	0.9 (0.2–2.3)	0.385
IgE, median (IQR), IU/mL	77.1 (11.1–132.2)	46.4 (9.2–160.6)	0.833
Subpopulation of lymphocytes, median (IQR), %			
CD19^+^CD23^+^	13.6 (10.5–19.4)	13.3 (9.9–18.1)	0.383
CD3^+^	61.3 (52.4–70.9)	65.1 (52.6–70.3)	0.626
CD3^+^ CD4^+^	35.0 (26.9–39.7)	35.3 (31.4–45.1)	0.063
CD3^+^CD8^+^	24.3 (17.6–28.6)	18.5 (15.6–26.2)	0.023
CD3^−^CD16^+^CD56^+^	9.6 (6.3–13.1)	8.9 (5.4–11.7)	0.647
CD3^−^CD19^+^	24.6 (18.2–31.1)	21.9 (17.6–30.6)	0.482
Humoral immunity, median (IQR), g/L			
Total IgA	0.43 (0.16–0.66)	0.32 (0.16–0.59)	0.273
Total IgM	0.89 (0.68–1.46)	0.9 (0.63–1.36)	0.700
Total IgG	6.09 (4.91–7.93)	5.73 (4.25–7.92)	0.254
Cytology of BALF, median (IQR), %			
Macrophages	50.0 (13.5–87.0)	54.0 (24.5–82.5)	0.554
Neutrophils	38.0 (10.0–77.5)	36.5 (12.0–62.0)	0.437
Lymphocytes	4.0 (2.0–9.5)	5.0 (2.0–6.7)	0.954
Eosinophils	0.0 (0.0–2.0)	0.5 (0.0–6.0)	0.027

*BALF* bronchoalveolar lavage fluid, *CMV* cytomegalovirus

There were no significant differences between patients with or without BALF CMV regarding any of the following: age; gender ratio; incidence of exclusive breastfeeding; ≥ 2 children in the household; peripheral blood eosinophils; CD19^+^CD23^+^, CD3^+^, CD3^+^ CD4^+^, CD3^−^CD16^+^CD56^+^ and CD3^−^CD19^+^ T cells; total IgE; total IgG; total IgA; total IgM; percentage of BALF macrophages; percentage of BALF lymphocytes, and; percentage of BALF neutrophils.

We further evaluated the correlation between the CMV DNA copy number (in BALF) and various continuous variables. We found that CMV DNA copy numbers (median 7560 (IQR 1200–71,150) copies/mL) were positively correlated with the duration of hospitalization (*r* = 0.33, *p* = 0.013), negatively correlated with age (*r* = − 0.41, *p* = 0.002) and with percentage of BALF eosinophils (*r* = − 0.38, *p* = 0.004).

None of the patients with positive BALF CMV in the present study received ganciclovir therapy. All of them recovered with symptomatic treatment.

## Discussion

The present study has demonstrated CMV replication to be highly prevalent among patients with severe recurrent wheeze. Test positivity was higher in patients aged < 12 months than in those aged 12 to 36 months and the median BALF CMV DNA copy number was higher in patients aged < 12 months than in patients aged 12 to 36 months. Our results were consistent with those of Cinel et al. [5], who also found an inverse relationship between age and BALF CMV PCR positivity. The immature immune system of young children might not be able to suppress CMV replication after primary infection, which offers a potential explanation for our observations of higher CMV DNA copy numbers in the younger age group of children with recurrent wheeze.

Our results also revealed that the frequency of CMV DNA detection in patients aged 12 to 36 months with a positive mAPI was higher than in patients aged 12 to 36 months with a negative mAPI. It has previously been suggested that patients with asthma might have impaired antiviral immunity [10], and it seems likely that such impaired antiviral immunity might also be exhibited by patients with a positive mAPI, leading to an inability to suppress CMV replication after primary infection.

The immunological basis of asthma involves an immune reaction mediated by T helper 2 (Th2) cells leading to chronic allergic inflammation of the airways due to infiltration by mast cells and eosinophils [11]. However, in the present study, we found that BALF CMV DNA copy numbers were negatively correlated with the percentage of BALF eosinophils. Furthermore, we also found a lower percentage of BALF eosinophils among patients who were BALF CMV positive, which was inconsistent with a hypothesis linking CMV infection with asthma-like Th2 inflammatory responses. Our findings in this respect are consistent with those of a previous study that utilized a murine CMV infection model of OVA-induced allergic airway disease in which the authors similarly reported a decrease in the BALF eosinophil count in the BALF CMV positive group, in addition to an enhanced mucus production independent of BALF eosinophils [12]. The impact of CMV infection on allergic airway disease needs further investigation.

In the present study, patients with positive BALF CMV had higher percentages of CD3^+^CD8^+^ T cells among their total T cell count than those with negative BALF CMV. This finding is consistent with those of a previous study [13]. Cytomegalovirus replication and reactivation are regulated primarily by cytotoxic T-cell immunity [1].

The median duration of hospitalization in the present study was longer in patients who were BALF CMV positive and was also positively correlated with CMV DNA copy numbers. This indicates that patients who were BALF CMV positive had more severe airway disease. Previous studies have found that CMV reactivation is associated with refractory responses to steroids and may result in disease progression in ulcerative colitis [14, 15]. However, studies concerning CMV reactivation and disease severity in patients with asthma or recurrent wheeze are lacking. The median duration of hospitalization of patients in the present study was 11 days and none of the patients with positive BALF CMV in the present study received ganciclovir therapy. Further studies are needed to address whether specific antiviral treatment would be beneficial for BALF CMV positive patients, especially in those patients with higher CMV DNA loads.

There are certain limitations associated with the present study. First, when performing the sample size calculation, we used a margin of error of 10%. A lower margin of error would have increased the statistical power of the study and reduced the probability of type II errors occurring. However, it would require a larger sample size than was achievable with the employed study design. Second, we only enrolled inpatients with severe recurrent wheezing, whilst a greater number of patients with mild to moderate wheeze were treated in the outpatient department. Therefore, the results cannot be generalized to patients with mild to moderate wheeze. Third, we did not perform basic research to explore the potential mechanisms by which the decline in BALF eosinophils may occur in patients with recurrent wheeze and concurrent CMV infection. Finally, we were unable to determine whether the higher prevalence of CMV DNA in the BALF of patients with recurrent wheeze resulted from virus reactivation as a consequence of airway inflammation or treatment with corticosteroids [16, 17]. Evidence suggests that both might be possible explanations.

## Conclusions

Despite these limitations, our findings suggest that CMV infection/reactivation in children with recurrent wheeze is associated with certain clinical characteristics of patients, including young age and a low BALF eosinophil count. In addition, higher CMV DNA copy numbers were associated with longer durations of hospitalization.

## Abbreviations

BALF: Bronchoalveolar lavage fluid
CMV: Cytomegalovirus
FFB: Flexible fiber optic bronchoscopy
IQR: Interquartile ranges
mAPI: Modified asthma predictive index
